# How do Australian maternity and early childhood health services identify and respond to the settlement experience and social context of refugee background families?

**DOI:** 10.1186/1471-2393-14-348

**Published:** 2014-10-06

**Authors:** Jane Yelland, Elisha Riggs, Sayed Wahidi, Fatema Fouladi, Sue Casey, Josef Szwarc, Philippa Duell-Piening, Donna Chesters, Stephanie Brown

**Affiliations:** Healthy Mothers Healthy Families Research Group, Murdoch Childrens Research Institute, Flemington Road, Parkville, Victoria 3052 Australia; General Practice and Primary Health Care Academic Centre, University of Melbourne, Parkville, Victoria 3052 Australia; Victorian Foundation for Survivors of Torture, 4 Gardiner Street, Brunswick, Victoria 3056 Australia; School of Population and Global Health, University of Melbourne, Parkville, Victoria 3052 Australia

**Keywords:** Afghan women and men, Refugee families, Social and mental health inquiry, Psychosocial, Maternity care, Early childhood health care

## Abstract

**Background:**

Refugees have poor mental, social and physical health related to experiences of trauma and stresses associated with settlement, however little is known about how refugee families experience maternity and early childhood services. The aim of this study was to explore the responsiveness of health services to the social and mental health of Afghan women and men at the time of having a baby.

**Method:**

Participatory methods including community engagement and consultation with the Afghan community and service providers in Melbourne, Australia. Bicultural researchers conducted interviews with Afghan women and men who had recently had a baby. Interviews and focus groups were also conducted with health professionals working in the region.

**Results:**

Thirty interviews were conducted with Afghan women and men who had recently had a baby. Thirty-four health professionals participated in an interview or focus group.

Afghan women and men reported significant social hardship during the period before and after having a baby in Australia, but were rarely asked about their social health by maternity and early childhood services.

Most health professionals recognised that knowledge and understanding of their client’s migration history and social circumstances was relevant to the provision of high quality care. However, inquiring about refugee background, and responding to non-clinical needs of refugee families was challenging for many health professionals. Factors that made it more difficult for health professionals to engage with Afghan families in pregnancy included limited understanding of the context of migration, dependency of many Afghan women on their husband for interpreting, short appointments, and the high likelihood of seeing different health professionals at each antenatal visit. Community-based maternal and child health nurses had more scope to work with interpreters, and build relationships with families, providing a stronger foundation for identifying and responding to complex social circumstances.

**Conclusion:**

There are significant challenges in providing comprehensive, high quality primary health care for Afghan families accessing Australian maternity and early childhood services. The limited capacity of public maternity services to identify families of refugee background and provide tailored service responses are contributing to inequitable maternal and child health outcomes for families of refugee background.

## Background

There is evidence that women of likely refugee background giving birth in Victoria, Australia have higher rates of stillbirth, fetal death in utero, and perinatal mortality compared with Australian born women [1, 2]. Women of refugee background are also less likely to attend the recommended number of antenatal check-ups, and more likely to attend emergency departments for obstetric complications. [1, 2]. Similar findings have been reported internationally [3, 4]. Refugee populations are also known to have higher risks of a range of physical, psychological and social health problems related to experiences of trauma, stresses associated with settlement in a new country and persistent disadvantage in the developed countries in which they live [5–8].

In the period 1998–2012 Australia’s Humanitarian Program, a program mainly for people recognised as refugees, was set at an annual intake of 13,750 and about a third of those granted visas under the Program settled in the state of Victoria. Over this time people who were born in Afghanistan formed the largest group of migrants to Victoria, with 4,843 settling under the Humanitarian stream and a further 1,400 arriving under the family-reunion scheme [9]. The Afghan population in Australia is predominantly young, and women are most commonly of child-bearing age.

Having a baby is a significant life event for all women and their families. For women and many men coming to Australia as refugees, pregnancy is often the reason for first sustained contact with Australian health services. Pregnancy check-ups and visits to early childhood services provide a recognised ‘window of opportunity’ for identifying and addressing modifiable social risk factors with potential long term benefits for maternal and child health.

There is accumulating evidence that exposure to stress and trauma during and preceding pregnancy contribute to a range of adverse outcomes for mothers and children, with potential to effect health across the life course [10, 11]. For families of refugee background stressful life events and social health issues encompass repeated traumatic events prior to arrival in Australia; circumstances associated with settlement such as poverty and low English proficiency; separation from other family members; and tension in changed spousal relationships. Mental health problems may be considered in this context of pre-arrival experiences and the stressors of settlement [12]. Given this level of hardship there are strong grounds for concern about likely impacts on the health and wellbeing of mothers and children.

The importance of assessing and responding to social and mental health risk factors for poor maternal and child health outcomes is receiving increasing attention, both in Australia and internationally. Several recent reports, including the Marmot Review in the UK, and the World Health Organisation’s Commission on Social Determinants of Health Report, Closing the Gap in a Generation, have identified investment in strategies to promote a ‘healthy start to life’ as having the greatest potential to reduce inequalities across the life course [10, 11, 13]. Australian perinatal guidelines, endorsed by the National Health and Medical Research Council, promote routine screening of women for mental health problems during pregnancy and the early postpartum period [14]. Antenatal care guidelines released by the Australian Government in 2012 recommend that all women, as early as practical in pregnancy, and again in the period 6–12 weeks after childbirth, are asked about stressful life events and social health issues, including past history of mental health disorders, availability of practical and emotional support, current or past abuse/violence, and current life events and major stressors [15]. The vulnerability of women from culturally and linguistically diverse backgrounds, and especially women of refugee background, is noted in the guidelines [15].

Whilst inequalities in health outcomes for families of refugee background are recognised by Australian health services, little is known about how families of refugee background experience Australian health care, particularly during and after pregnancy. Several Australian studies have investigated immigrant women’s experiences of maternity care [16–18]. However, there is limited research focusing on how families of refugee background, including men, navigate Australian health care, or about the responsiveness of health services to the social circumstances around forced migration and settlement. Likewise, there have been no major studies exploring the experience of health professionals and other service providers who support refugee families at the time of having a baby.

Identifying that families accessing maternity and early childhood services have a refugee background is a fundamental first step in tailoring health care to the needs of this specific population. However identification of families of refugee background is known to be difficult [19]. This paper examines how health professionals working in maternity and early childhood services identify refugee families in their care, inquire about a range of social health factors relevant to families of refugee background, and respond to social health issues as part of routine care. Specifically, the paper aims to:

1. investigate Afghan women and men’s experience of the way that health professionals approach inquiry about social factors affecting families having a baby in a new country, and
2. investigate how health professionals identify and respond to the settlement experience and social context of families of refugee background.

## Methods

### Setting

The study was conducted between 2012 and 2013 in the local government areas of Greater Dandenong and Casey in Victoria. These large outer suburban regions of Melbourne are major areas of settlement of refugees in Australia. The City of Greater Dandenong has the highest number of settlers in any Victorian municipality. Of the 2,737 recently-arrived migrants settling in this local government area in 2012/13 nearly a third were humanitarian entrants, largely from Afghanistan, Sri Lanka, Pakistan and Iran. At the time of the study a quarter of all refugees in this region of Melbourne were from Afghanistan, including a significant number of people who had arrived in Australia within the last five years.

Several services provide maternity and maternal and child health care in the region. One large health network includes three maternity hospitals providing care to over 8,000 women each year. Around 10% of these women come from humanitarian source countries. Public antenatal care is offered to women and their families through public hospital antenatal clinics where clients are likely to see different midwives and/or doctors at each pregnancy visit. Alternatively, families can attend community-based practitioners (usually general practitioners), or access care via shared care arrangements where care is shared between a community-based general practitioner (GP) and hospital antenatal clinic. Irrespective of the model of pregnancy care, women booked as public patients have labour and birth care and care in the days following birth provided by rostered hospital staff.

The Maternal and Child Health Program in Victoria is a universal service available to all families with children up to school age. The service provides regular child health and developmental checks, screening and referral to additional services if required, and assessment and support related to maternal health. Care is provided by multi-qualified maternal and child health nurses and is organised around ten key consultations, including a home visit shortly after birth and a further six visits in the infant’s first year. Families are registered with a centre closest to their home and are likely to see the same nurse at each visit. Other services include the Refugee Health Nurse program that responds to the often complex health needs of arriving refugees [20], and the Healthy Mothers Healthy Babies service that assists pregnant women requiring additional support due to significant social adversity [21].

### Data collection

Data collection and study methods were informed by community and service provider consultations that preceded the design of the study. Separate community and stakeholder advisory groups were established to guide and advise the research team on appropriate consultation strategies. Community advisors facilitated consultation with around 100 members of the Afghan community. Stakeholder advisors included health service managers, clinicians and government policy planners.

### Recruitment and interviews

#### Afghan women and men

Women and men who were born in Afghanistan, aged 18 years or older, and who had a baby that was around 4–12 months of age were eligible to participate in the study. Purposive recruitment methods and multiple initial contacts were used to optimise recruitment and ensure diversity of potential participants. Recruitment was undertaken by two community researchers (FF, SW) who worked with the community advisors and organisations that had assisted with the community consultation, in order to invite women and men to participate in an interview. A postcard with information about the study and details about how to take part, in Dari and English, was distributed to local groups and services, and the postcard was printed in the Afghan community newspaper.

Potential participants were provided with a telephone number to contact the community researchers to register their interest in participating in an interview. Upon contact women and men were provided with verbal information about the study and handed or mailed a copy of the project information in Dari or English. Participants were asked to consent to the research, either in writing or verbally.

Individual interviews were conducted by the community researchers and undertaken in the language preferred by the participant, usually Dari or English. Participants were also asked their preference for where the interview should take place. The majority of interviews were undertaken at Foundation House, were audio-taped with the permission of participants, and on average took around 60 minutes. Participants were given a $50 supermarket/department store voucher to thank them for taking part.

#### Afghan women and men: interview schedule

The interview schedule was designed by the partnership team drawing on the issues raised during the community consultation (e.g. men’s attendance at antenatal check-ups; gender of providers) and questions asked in a 2008 population-based survey of recent mothers [22–25]. The themes covered in the semi-structured interview schedule were similar for women and men (see Table 1 for themes covered in the interview schedule for women). Modification of the schedule took place following piloting with six members of the Afghan community, and was translated into Dari by the community researchers.

**Table 1:** **Interview schedule for Afghan women and men**

**About you and your family**	• Date of birth (Q)
	• Country of birth; transit country; year of arrival in Australia (Q)
	• Hazara, Pashtu or Tajik background (Q)
	• Other children: age & country of birth (Q)
	• Household composition (Q)
	• Schooling (Q)
	• English proficiency; literacy in own language
	• Health care concession card (Q)
	• Paid employment (Q)
	• Transport (Q)
**About your pregnancy care**	• Gestation at first antenatal check-up (Q)
	• Knowledge of health system; accessing care
	• Who provided care; location of care; getting to care
	• Husband’s attendance; role at visits
	• Interpreter required; experience of language support
	• Attendance at emergency departments
	• Experience of screening tests; explanation of results
	• Interactions with care providers
	• Knowledge and attendance at childbirth education
	• Information about pregnancy and maternity care
	• Good things about care; what could have been better
**Having your baby**	• Place of birth (Q)
	• Arrival at hospital
	• Family/friends present
	• Language: who interpreted; experience of language support
	• Care providers: interaction; explanations
	• Mode of birth (Q)
	• Good things about care; what could have been better
**After the birth**	• Length of stay; reason for going home
	• Overall experience of hospital care
	• Infant feeding; support for breastfeeding issues
	• Home visits following birth
	• Interpreter use; experience of language services
	• Experience of Maternal and Child Health Services: access; continuity; liked/didn’t like about care; what could have been better
	• Visits to General Practitioners
**Psychosocial issues**	• Asked about: relationship problems; feelings of sadness or depression; your family here and overseas; financial worries; violence at home; housing problems; legal problems (Q)
	• Experience of being asked about things happening in life
**Support during and after pregnancy**	• Accessing information about own health & child health
	• Knowledge and use of local services (e.g. refugee specific services; playgroups; legal services) for self and husband (Q)
**Overall views of care**	• Asked about & able to follow traditional practices
	• Perceived discrimination (e.g. talked down to, treated unfairly) (Q)
**Role of men**	• Life as an Afghan man in Australia
	• Role as a father in Australia compared with Afghanistan

(Q): Quantitative item.

Women and men were asked if health professionals had asked them about a range of mental health and social health issues (e.g. if they felt sad or depressed, were experiencing relationship problems, or had problems with housing). Participants were also asked whether a midwife/nurse or doctor inquired about emotional health and things happening in their lives, and whether the health professional responded by offering support. Participants who reported that they had not been asked about their mental health or what was happening in their lives, were asked whether they would have liked health professionals to ask them about these issues.

#### Health professionals

Health professionals were identified using several approaches. Initial direction was provided by the sector stakeholder advisory group that was established at the beginning of the project. During the community consultation phase, several agencies were identified as key for the Afghan community. These agencies were contacted, key informants identified and invited to participate in the study. Participants were then asked to identify health professionals with further invitations made to these people. All interviews and focus groups with health professionals were conducted by one of the authors (ER) with the exception of one conducted by JY.

#### Health professionals: interview and focus group schedule

The interview and focus group schedule for health professionals, as outlined in Table 2, focussed on their practices of working with and responding to the needs of Afghan families. Informants were asked what they consider the main health and social issues for the families that they see; if they assess for social and emotional well-being of their Afghan clients, and how they respond if concerns are identified.

**Table 2:** **Interview schedule for health professionals and community workers**

**Current position**	• Organisation, role, length of time in role
**Experience of working with Afghan/refugee community**	• Frequency, number of clients
	• Identifying refugee background
	• Perspective of health, social and cultural issues in community
	• Patterns of service utilisation of clients
	• Perspectives of male and female roles at the time of having a baby and after birth
**Assessments, screenings conducted with clients**	• Assessments conducted: social and emotional wellbeing
	• Identification of what is happening in Afghan clients’ lives
	• Referrals: are they made; to where?
	• Continuity of care provided
	• Practices developed to respond to the needs of Afghan and refugee background families
**Interpreter use**	• Identifying need for interpreter
	• Current uptake, any issues
	• Bicultural workers
**Information and advice provided to clients**	• Access to appropriate translated information
	• Perspectives of understanding/adherence of advice given
	• Unmet needs
	• Other information/resources required
**Ways of working better**	• Support for health care professionals
	• Support for community to access/engage with services
	• Working with other agencies
	• Organisational change to support working with refugee families

### Analysis

The majority of interviews (80%) were conducted in Dari or another Afghan language. Interviews conducted in community languages were translated and transcribed into English by the community researchers. Once transcribed, the community researchers cross-checked each other’s transcription documents by listening to the audio recordings and discussing any amendments. NVivo10 [26] was used to store and manage interview data.

A series of quantitative questions were asked of all community participants (see Table 1). Due to the sample size, simple descriptive frequencies for these items were conducted. For all open ended questions thematic analysis was completed [27]. Analysis began after the first three interviews with women which were coded, informing the coding manual. A coding manual was developed using some a priori codes from the interview schedule; an iterative process was used to add additional codes to the manual (undertaken by ER, JY, FF,SW). This coding manual was used to code all women and men’s interviews. JY and ER cross-checked the coding of all interview transcripts, providing an opportunity to discuss differences in the interpretation of the data. Codes were then grouped into logical categories which then provided the overarching themes. Not all themes are reported in this paper.

The community researchers’ active role in the data analysis enhanced the reliability and validity of the findings by providing their cultural knowledge and insight. Concurrent coding, discussion and interpretation of the findings amongst the project team enhanced the trustworthiness of the collected data.

All interviews and focus groups with health professionals were digitally recorded, transcribed by an external agency, and cross-checked for accuracy of transcription. All transcripts were read (by ER, JY) and imported and stored in NVivo10 [26]. Coding and categorising of data was undertaken (by ER), and key themes identified. For the purpose of analyses presented in this paper, ‘mental health issues’ include psychological health issues affecting both women and men. ‘Social health issues’ encompasses a diverse range of issues including: exposure to repeated traumatic events, separation from family members, low English language proficiency, poverty, housing problems, tensions in partner relationships and family violence.

Quotes have been selected to illustrate the themes. Community participants have been identified by gender. Health professionals have been identified by profession (midwife, maternal and child health nurse, medical practitioner) or as a community-based care provider (for the small group of informants whose role primarily involved providing care to families of refugee background e.g. refugee health nurse).

### Ethics

The project was approved by the research ethics committees of the Victorian Foundation for Survivors of Torture and the Royal Children’s Hospital.

## Results

### Participants

#### Afghan women and men

Sixteen woman and fourteen men participated in an interview when their infant was 4–12 months of age. Three couples participated in six individual interviews. Table 3 outlines the characteristics of participants. All were born in Afghanistan; two-thirds were of Hazara background reflecting trends in settlement of Afghan people under Australia’s Humanitarian Program. Half of the participants had been in Australia for five years or less, with all but three having lived in other counties before arriving in Australia.

**Table 3:** **Characteristics of Afghan participants (n = 30)**

	Number
**Background**	
Hazara	21
Tajik	6
Pashtu, Afghan or Sadath	3
**Length of time in Australia**	
<12 months	3
1-2 years	6
3-5 years	6
6+ years	11
**Country lived in before coming to Australia**	
Afghanistan	3
Pakistan	23
Iran, Syria, United Arab Emirates	4
**Language spoken at home**	
Dari only	12
Hazaragi, Pashto, Urdu, Arabic	11
Dari & other language	5
Dari & English	2
**Education**	
Completed secondary school	15
Did not complete secondary school	15
**Number of children**	
One child	10
Two or more	20
**Household**	
Nuclear	20
Extended	10
**Health care concession card**	
Yes	26
No	4

Of the 14 Afghan men, half were unemployed. One of the 16 women was employed part-time. The majority of participants were holders of health care concession cards, providing access to free or low cost government funded health care services and pharmaceuticals. Of the 11 women who had had a baby before, five were giving birth in Australia for the first time. All participants spoke an Afghan language at home. Five participants noted that they were not literate in their community language. Of the 16 women, five said their English was “good, six reported that their English was “OK” and five didn’t speak English at all or not well. Nine of the 14 men reported that their English was “good”.

At the time of the interviews twenty of the participants were living in nuclear households and ten with extended family, including participant’s siblings, their partners and children (Table 3).

#### Health professionals

Thirty-four health professionals participated in interviews and focus groups. Participants included midwives, general practitioners (GPs), refugee health nurses, maternal and child health nurses, obstetricians, other health care providers and community bicultural workers. All provided care to families of refugee background including Afghan clients. This ranged from seeing one Afghan family a week to seeing up to five families a day.

### Language services in the context of care

All Afghan participants received care in the public maternity system, two-thirds through public antenatal clinics and a third with general practitioners. Around three-quarters of Afghan men and women taking part indicated that they required language services when using maternity and early childhood services. There was some diversity in women’s experiences of language services during pregnancy check-ups with many noting that their husband interpreted for them. Very few reported having access to professional interpreter at *all* antenatal visits. Most women had interpreter-mediated appointments for the first home visit with a midwife or maternal and child health nurse after discharge from hospital, some with a telephone interpreter.

Gender of the care provider and language support were frequently raised in the context of participant’s experience of care. For many the gender of health professionals and interpreters was critical to how women engaged with care, including their comfort in disclosing health or family concerns.

*My wife … wanted the interpreter to be a female but he was male. It was very shameful anduncomfortable for my wife. She told me she asked for a female interpreter but they didn’t listen to her. She couldn’t tell her personal problems to a male interpreter as in our culture it’s not right. She was very upset about that.* (Male participant)

### Women and men’s experience of being asked about social health issues

Table 4 reports women’s responses to a series of questions asking about their contacts with health professionals during pregnancy and since the birth of their baby. Around two-thirds reported that a doctor, midwife or maternal and child health nurse asked about their family in Australia and overseas, six reported being asked if they had relationship problems with their husband, and three reported being asked about violence at home. Very few women were asked about housing, legal and financial concerns. Overall women reported that maternal and child health nurses were more likely to ask about social health issues such as housing and financial problems and family relationships, than midwives and GPs.

**Table 4:** **Number of Afghan women (n = 16) reporting that a health professional asked about social circumstances during pregnancy or after the birth**

	Doctor asked	Midwife or nurse asked	No-one asked
About your family here in Australia and overseas?*	4	9	6/16
If you had relationship problems?*	1	6	10/16
**If you had financial worries?**	0	3	13/16
**If you had housing problems?**	0	2	14/16
Whether there was violence at home?*	1	3	13/16
**If you or anyone at home had legal problems?**	0	1	15/16

*Total number >16 when women reported both a doctor and midwife/nurse asked.

All of the men had been in contact with health professionals whilst attending appointments with their wives at some point during and/or after pregnancy. Men were far less likely to be asked about their concerns at these appointments. Of the 14 Afghan men, four were asked about their family and three were asked if they felt sad or depressed. Only one man was asked about financial worries. None were asked about housing or legal problems.

Participants were asked whether they would have liked to be asked about things happening in their lives, and if they had received help with issues such as housing problems or financial worries. Several participants indicated that they would have liked health professionals to ask about these issues.

*Yes [would have liked to have been asked], we started our life from zero, and [wanted information] on how to start work.* (Male participant)

*I would have liked them to ask me about my parents and other things in my life that concerns me, but I cannot encourage myself to initiate the conversation.* (Female participant)

*Yes I wanted to be asked … I wanted to know what to do if any of those problems occurred – where should I get hep if something occurred?* (Male participant)

Many, however, didn’t feel that inquiry about social health issues was the role of the health professional.

*Yes I can talk to them [health professionals in pregnancy] about my health problem but don’t feel comfortable sharing with them my personal and domestic affairs … one thing I understand is that it is not part of their job to listen to our family problems.* (Female participant)

Most saw social health issues and family life as private and personal matters best discussed with other family members rather than health professionals. Women reported that their husbands and/or mothers were the family members they were most likely to discuss ‘social’ issues with. Some noted the limitations of raising these issues with extended family and other community members particularly in relation to confidentiality, concerned that problems disclosed could become the topic of “community gossip”.

How questions were asked and who was in attendance at the time influenced the way that participants responded to questions about social issues.

*… the nurse was always just asking my wife if she had any problems with me. I didn’t like that because that itself creates a problem between wife and a husband. I said they should also ask the male if they have any problems. You could make a problem between family if they don’t have a problem … when someone discuss their family problem with others the problem become worse, then [hard] to be solved.* (Male participant)

Yet the majority of participants who reported that a health care professional had inquired about social issues noted that this had been helpful.

*The midwives and nurses were the best emotional supports for me during the entire time because I was going through the toughest phase of my life. The maternal and child health nurse calls me at home to check that everything is OK … she has not only been a verbal support for me but has linked me with support services.* (Female participant)

### Identifying and responding to social health issues: the experience of health professionals

#### Identification of refugee background

Most health professionals could identify their clients were Afghan or of Afghan descent as their country of birth and language spoken was recorded on client records. With the exception of those working in refugee-specific services, it was difficult for health professionals, from an organisational perspective, to identify that clients had a refugee background. While recording country of birth, and language spoken, was standard practice in many settings, there was no standard procedure for asking about, or recording, whether a client had a refugee background.

Without formal identification processes some health professionals, mostly community-based providers, reported ways that they identified if their client had a refugee background. Informal questions were seen as preferable to asking directly about refugee-background.

*I naively thought…it was because they all had the birth date the 1st of the 1st, I thought oh yeah I can tell. But now…I actually ask them, how did you come to Australia, what was your journey and find that out. And they’re actually really interested that you ask.* (Maternal and child health nurse)

*We ask them about what sort of accommodation they’re living in, what they and their partner’s employment status is, often then it comes out if they’re not working or they’re on benefits or whatever the situation is, but we don’t specifically ask them if they’re refugees.* (Midwife)

Many felt that understanding the context of women’s experience in coming to Australia was important to the provision of care.

*As far as thinking about their pregnancy care and then also for baby, yeah you’d want to know what circumstance they’ve come from, what they’ve been exposed to as far as physical and sexual violence, and if they have any finances ‘cause obviously that’s important for preparing for a baby, if they have any family here, if they’re on their own because that’s obviously going to impact on how they care for the baby.* (Midwife)

*It’s the extended family [in Afghanistan] that actually care for that family. So you take that away and there are refugees that come to Dandenong that have never ever parented before and they might have two or three kids but they have never truly parented because they’ve had their community, they’ve had their family who have been there … loneliness and isolation … you’ve got to put it into the context [of being a refugee from Afghanistan].* (Maternal and child health nurse)

In contrast, some health professionals believed that identifying whether their client was of refugee-background was not important, as it would not alter the course of care that provided.

*I suppose we tend to treat everyone pretty much the same …it doesn’t really make a big difference to what we can offer them.* (Medical practitioner)

Others felt that asking clients about their refugee background could result in disclosure of information that they did not feel they could respond to and deal with directly, such as mental health issues. One health professional suggested that not all clients would want to be identified as ‘refugee’ per se, suggesting that it was more important to identify other aspects of their lives that could indicate particular needs and supports required.

*“Getting a picture of what’s going on in this family”: providers’ views*

How health professionals approached assessment of social health issues and mental health varied considerably in different settings. Public maternity hospitals in the region ask women to self-complete a form at the time of the pregnancy booking visit asking them about their emotional health and family violence. For women with limited English proficiency this was reportedly completed with the assistance of their husband or a professional interpreter. Some hospital staff talked about not feeling particularly confident about their skills in talking with refugee women about the issues identified on the psychosocial assessment form or knowing how to respond if women disclosed family violence or a history of depression. The idea that *‘nothing can be done for them’* stopped some health professionals embarking on assessment, except when there was an obvious crisis situation. Others felt that the assessment form had limited utility for women of refugee background, and unless women initiated conversation about what was happening in their life, formal assessment was not undertaken.

*The difficulty is when you do meet ladies and you think “yeah, you are very flat” so they are not making a lot of eye contact, not really answering questions, their head is down, I’m not getting any connection or any information from them. It makes you wonder what’s going on. But to be honest we don’t do it [psychosocial assessment], unless there was anything concrete that they told you - it’s not really dealt with.* (Midwife)

Maternal and child health nurses who are encouraged to use an assessment tool, such as the Edinburgh Postnatal Depression Scale [28] to assess mental health, noted that they rarely used it as prescribed, often adapting it because words in the scale were difficult to explain to women.

*The Edinburgh Postnatal Depression Scale - I must admit that I don’t use that … I ask them things like how they are feeling in their heart, I ask them are they sleeping, are they eating, do they cry a lot …and dad’s will say ‘oh she cries all the time.’* (Maternal and child health nurse)

It was more common for early childhood nurses to ask women and men what was happening in their lives as indicators of their mental health.

*Always ask them how they are, and I’ve learnt to ask the questions differently now because postnatal depression is not a concept that Afghan women understand but feeling very sad and missing your family and then asking about other things.* (Maternal and child health nurse)

Several participants detailed the steps they take in establishing a rapport with women and the importance of this in terms of inquiring about their migration history and social circumstances.

Clinicians who saw the same family for antenatal or early childhood health visits noted the benefit of continuity of caregiver in the development of a trusting relationship, developing an understanding of a woman’s needs, and a woman’s comfort in disclosure.

*Well I think it’s a relationship that you build, listening to them, finding out a little bit how they see things and see the world. I’ve found its good having a bit of an understanding about their cultural background. So I’d say it’s more the relationship that is most helpful in working with refugee families.* (Maternal and child health nurse)

*And obviously the most unfortunate thing about the service is that lack of continuity of care. If you saw someone regularly then you wouldn’t ask the same questions [every time] and I think the documentation for emotional health is very little. So for us to be able to communicate what happened at each visit is almost impossible, we start again …* (Midwife)

The component of social health assessment that was most challenging for health professionals was asking women about family violence. For maternal and child health nurses family violence was seen as a ‘big’ issue for women, irrespective of social background. Antenatal providers were less likely to report asking Afghan women about family violence unless women raised this as an issue themselves. The presence of husbands at appointments; recognition of women’s concerns about confidentiality particularly in the presence of an interpreter who may be known in the community; and an understanding of women’s relationship with her husband in the context of the refugee and settlement experience, were cited as barriers to inquiry.

*The women do not want to [disclose domestic violence] because their husband is everything and they do not want to because they totally depend sometimes on their husband. If they do not have their husband there by their side they do not feel comfortable, like how to get food, how to do other things - they think they will not survive without their husbands so that’s why they do not disclose.* (Community-based care provider)

Getting to know the woman and her family, fostered by visiting women in their homes and seeing the same provider at antenatal and postnatal visits, were identified as particularly helpful in assessing family violence over time.

Providers described the importance of putting people at ease, being friendly, welcoming and showing an interest in people’s lives. Several community-based providers noted that whilst these factors were important for all families, understanding the refugee experience particularly trauma and the issues related to settlement, was essential in responding to families of refugee background. The latter was commonly cited as important to assessing social health and wellbeing particularly in opening up conversations about issues that women may not consider part of antenatal care.

## Discussion

### Social and economic determinants of health

The demographic profile of Afghan women and men participating in the study reflects the challenges of forced migration and settlement in a new country. All but three of the participants had spent time living in another country, mostly in Pakistan, prior to coming to Australia. All spoke an Afghan language at home and most did not speak English well. Half of the men were unemployed, and of those employed most were working in a part-time capacity. Nearly all families had health care concession cards, an indicator of financial disadvantage. The majority lived in nuclear households irrespective of how long they had been in Australia. Whilst this profile is aligned with what is known of other humanitarian entrants to Australia [29], the social circumstances of these families even years following settlement is striking. The circumstances of settlement combined with the trauma of fleeing from one’s country of origin and seeking refuge elsewhere are important considerations for those providing health care, particularly at the time of a major life event such as having a baby.

### Identification of refugee background as a first step in providing health care

Being aware of a client’s migration experience and the social context of their lives, particularly if they have a refugee background, can assist health professionals to tailor care to meet specific needs. At the time of the study there was no agreed or standard way for health professionals working in maternity or early childhood services to identify people with a refugee background.

The level of social adversity experienced by the Afghan participants combined with their reports of being infrequently asked by health professionals about what was happening in their lives suggest that health care providers face a number of challenges in responding to social health issues. These challenges are likely to be multifaceted, reflecting the complexity of the Australian health care system and the demands on the maternity sector. Our findings also suggest that many health professionals have limited understanding of the refugee experience and how best to respond to social and emotional health, and issues such as family violence, in this context.

Whilst many health professionals interviewed gave examples of the ‘social’ questions that they ask families, including whether women and men have the support of family and friends, very few health professionals explored the migration history of those in their care. Indeed some providers queried why knowing that a family was from a humanitarian source country was important. Others suggested that even if they knew a client had come to Australia as a refugee, it wouldn’t make any difference to what they could offer by way of care.

### Identifying and responding to the settlement experience

This paper has described the responsiveness of health care providers to Afghan women and men’s social health rather than quantify social stressors or mental health issues per se. Several studies contribute to a body evidence about the higher rates of depression after birth amongst migrant women of non-English speaking background compared to native born women [30]. Factors that contribute to poorer mental health include social isolation, separation from extended family and the stresses associated with settlement [18, 31–34].

Some Afghan men and women didn’t expect health professionals to inquire about social issues. On the other hand when maternal and child health nurses asked, this was welcome. Maternal and child health nurses noted that developing a relationship with a woman and her family helped in understanding social circumstances and how best to inquire about the migration, settlement experiences and social wellbeing. Longer appointment times, frequency of key visits particularly in the first 12 months following birth, and the likelihood of families seeing the same nurse at each visit facilitate the development of relationships between the community-based nurses and their clients. Accessing community-based maternal and child health services close to home overcomes some of the issues identified by the Afghan families about reliance on husbands to provide transport to hospital-based appointments. The fact that some Afghan women participating in the study reported visiting a known maternal and child health nurse on their own (i.e. without their husband being present) is likely to explain, in part, the finding that maternal and child health nurses more commonly asked about social circumstances, including family violence.

Current models of providing public antenatal care in Australia pose particular challenges in relation to how inquiry regarding stressful life events and social health issues can be integrated into routine care. Australian maternity care in busy public hospital clinics is characterised by short consultations, the likelihood that women will see different midwives and medical practitioners at each visit, limited capacity to ensure access to female caregivers and difficulties in accessing interpreters. There is little system flexibility to allocate time for interpreter-mediated appointments [35], limited access to bicultural workers or capacity to work with local community services.

The lack of information and resources to assist women and their families understand the unfamiliar Australian health system including what to expect from maternity care and the role of the various providers has also been reported by others [36, 37]. Limited information, the challenges facing the maternity sector, coupled with the dependency of Afghan women on husbands for getting to services and for interpreting, constrains antenatal clinic staff in responding to issues such as family violence.

### Strengths and limitations

Major strengths of this study include the involvement of women and men of refugee background, and participatory processes to seek community and service provider input into design and conduct of the study. People of refugee background are often excluded from mainstream research, and men, irrespective of background, are infrequently asked about their experiences of maternity and early childhood health services. A particular strength of the study was the engagement with community through a substantial community consultation, and the involvement of the project’s Afghan community advisors. This shaped the research phase with Afghan women and men identifying issues to canvass in the research that were important to the community, and providing direction to the research team about culturally appropriate approaches to data collection and the sharing of results.

The two components of the study - interviews with members of the Afghan community and with health care providers - provided rich data on the views and experiences of consumers and providers of maternity and early childhood health services. The breadth and relevance of the findings to those engaged in caring for families has been well received, providing an indication of where efforts need to be placed to improve quality and outcomes of care.

On the other hand, as an exploratory study designed to examine experiences of care across pregnancy, labour and birth, and the postnatal period, we were limited in the extent to which we could explore issues in detail. Other limitations of the study include: the inclusion of only one community group, in one region of Melbourne. The findings may not be generalisable to other community groups, or to services in other parts of Melbourne, or other urban centres in Australia. However, in presenting the findings and discussing the results with service providers and organisations working with families of refugee background, it is clear that the stories we were told resonate with experiences of other groups, in other settings.

### Implications for maternity and early childhood health services

How services identify families of refugee background is fundamental to planning of health services and care provision. Our findings support calls for standardised procedures to improve identification of people of refugee background in clinical settings.

Better ascertainment of refugee background is a first step in improving care. Our findings show that some health professionals still question the need for finding out about refugee background, and the capacity of the system to support them in providing care to families with complex needs. Building an understanding of the refugee experience, what health care providers need to be mindful of in providing care to families of refugee background, and knowledge of services for referral, is likely to go some way in building workforce capacity to assess and respond to the social circumstances of refugees. Interactive training opportunities incorporating knowledge of the refugee and asylum seeker experience and ways of working with these families is a strategy to enhance health professionals understanding and skills.

Care tailored to vulnerable families is constrained, or enabled, by the way health services are structured, care is organised and the availability of resources. Whilst exploring this in detail was not within the scope of the study, both Afghan participants and health care providers indicated factors that impacted on their experience of care including for example the time available for interpreter mediated appointments, and impact of seeing different care providers in pregnancy. Any attempts to improve the responsiveness of health services to the needs of families of refugee background need to consider innovative ways to work within system constraints.

## Conclusion

There are complex challenges in providing comprehensive, high quality primary health care for families of refugee background accessing Australian maternity and early childhood services. The limited capacity of public maternity services to identify families of refugee background and provide tailored service responses, including access to interpreter-mediated appointments, and continuity of caregiver, are contributing to inequitable maternal and child health outcomes for families of refugee background.
